# Aqua­{μ-*N*-[3-(dimethyl­amino)­prop­yl]-*N*′-(2-oxidophen­yl)oxamidato(3−)}(1,10-phenanthroline)dicopper(II) nitrate

**DOI:** 10.1107/S1600536810035919

**Published:** 2010-09-11

**Authors:** Zhongjun Gao, Yanbao Wang

**Affiliations:** a Department of Chemistry, Jining University, Shandong 273155, People’s Republic of China

## Abstract

The title complex, [Cu_2_(C_13_H_16_N_3_O_3_)(C_12_H_8_N_2_)(H_2_O)]NO_3_, consists of a nitrate ion and a binuclear Cu^II^ unit in which the oxamide ligand has a *cis* geometry, is fully deprotonated and acts in a bidentate fashion to one Cu^II^ atom and in a tetradentate fashion to the other Cu^II^ atom. The Cu^II^ atom coordination geometries are distorted square-planar and distorted square-pyramidal. In the crystal structure, binuclear complexes and nitrate ions are connected by classical O—H⋯O and non-classical C—H⋯O hydrogen bonds into a three-dimensional framework. The alkyl chains of the anion are equally disorded over two positions.

## Related literature

For background to oxamide-bridged transition metal complexes, see: Kou *et al.* (1999 ▶); Ojima & Nonoyama (1988 ▶). For a related structure, see: Wang *et al.* (2003 ▶).
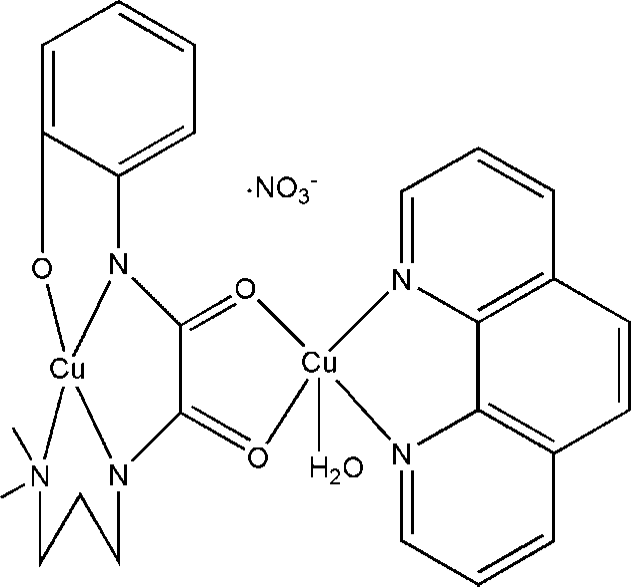


## Experimental

### 

#### Crystal data

[Cu_2_(C_13_H_16_N_3_O_3_)(C_12_H_8_N_2_)(H_2_O)]NO_3_
*M* *_r_* = 649.60
Triclinic, 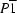

*a* = 10.543 (2) Å
*b* = 11.070 (2) Å
*c* = 11.404 (2) Å
α = 89.88 (3)°
β = 82.28 (3)°
γ = 78.24 (3)°
*V* = 1290.7 (4) Å^3^
*Z* = 2
Mo *K*α radiation
μ = 1.71 mm^−1^
*T* = 296 K
0.56 × 0.51 × 0.46 mm

#### Data collection

Bruker SMART CCD diffractometer
Absorption correction: multi-scan (*SADABS*; Sheldrick, 1996 ▶) *T*_min_ = 0.448, *T*_max_ = 0.508
12596 measured reflections
5984 independent reflections
4281 reflections with *I* > 2σ(*I*)
*R*_int_ = 0.022

#### Refinement

*R*[*F* ^2^ > 2σ(*F* ^2^)] = 0.042
*wR*(*F* ^2^) = 0.129
*S* = 1.00
5984 reflections
397 parameters
24 restraints
H-atom parameters constrained
Δρ_max_ = 0.62 e Å^−3^
Δρ_min_ = −0.36 e Å^−3^

### 

Data collection: *SMART* (Bruker, 1998 ▶); cell refinement: *SAINT* (Bruker, 1998 ▶); data reduction: *SAINT*; program(s) used to solve structure: *SHELXS97* (Sheldrick, 2008 ▶); program(s) used to refine structure: *SHELXL97* (Sheldrick, 2008 ▶); molecular graphics: *SHELXTL* (Sheldrick, 2008 ▶); software used to prepare material for publication: *SHELXL97* and *PLATON* (Spek, 2009 ▶).

## Supplementary Material

Crystal structure: contains datablocks I, global. DOI: 10.1107/S1600536810035919/jj2053sup1.cif

Structure factors: contains datablocks I. DOI: 10.1107/S1600536810035919/jj2053Isup2.hkl

Additional supplementary materials:  crystallographic information; 3D view; checkCIF report

## Figures and Tables

**Table 1:** Selected bond lengths (Å)

Cu2—O2	1.938 (2)
Cu2—O3	1.967 (2)
Cu2—N5	1.986 (3)
Cu2—N4	1.998 (3)
Cu2—O4	2.275 (2)
Cu1—N1	1.924 (3)
Cu1—O1	1.950 (2)
Cu1—N2	1.976 (2)
Cu1—N3	2.007 (3)

**Table 2:** Hydrogen-bond geometry (Å, °)

*D*—H⋯*A*	*D*—H	H⋯*A*	*D*⋯*A*	*D*—H⋯*A*
O4—H4*A*⋯O1^i^	0.91	1.84	2.745 (3)	172
O4—H4*B*⋯O5	0.89	1.97	2.839 (5)	164
C19—H19⋯O4^ii^	0.93	2.51	3.355 (5)	152
C21—H21⋯O5^ii^	0.93	2.53	3.437 (7)	167

Symmetry codes: (i) 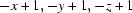; (ii) 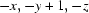.

**Fig. 1.**
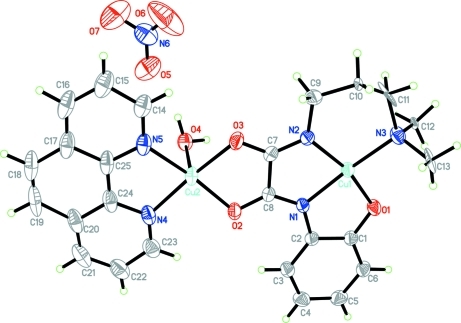
The molecular structure of C25H25N6O4Cu2+, NO3- with 30% displacement ellipsoids. Symmetry code as in Table 2.

**Fig. 2.**
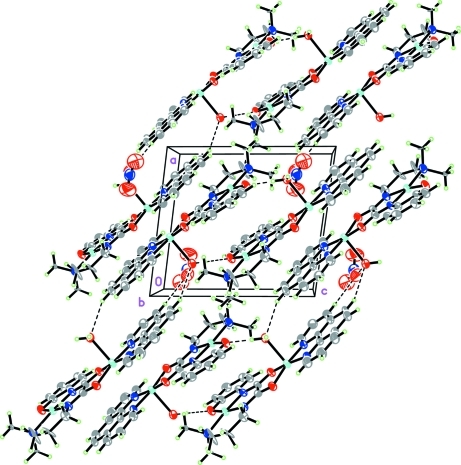
Packing diagram for C25H25N6O4Cu2+, NO3-. The O—H···O and C—H···O hydrogen bonds are shown by the dashed lines.

## supplementary crystallographic information

### Comment

Much research effort has been dedicated to studying oxamide-bridged transition metal complexes because of their bioactivities and the versatile bridging function (Kou *et al.*, 1999; Ojima & Nonoyama, 1988).

The title compound, C_25_H_25_N_6_O_4_Cu_2_^+^, NO_3_^-^is a binuclear copper(II) complex and the structure is similar to that seen previously in a resemble compound (Wang *et al.*, 2003)(Fig. 1).  In the dinuclear cation, the oxalate groups bridge the two copper(II) ions. The separation of copper atoms is 5.192 (2) Å. The Cu-atom coordination geometries are regarded as distorted square and square pyramid, respectively.  The oxamide ligand has a cis geometry, is fully deprotonated and acts in a hexadentate fashion.  Cu—O and Cu—N bond lengths are shown in Table 1.  For Cu1, the four atoms (O1, N1, N2, N3) from the oxalate groups build the square plane.  The average value of the copper to N1, N2 and N3 bond distance is 1.969 Å. For Cu2, the donors on the oxamide (O2, O3) and the phen (N4, N5) offer the basal plane and the oxygen of a water molecule occupies an apical position with a bond length of 2.275 (2) Å.  The maximum displacement from the least-square plane is 0.0055 (2) Å for O2 and the Cu2 atom lies 0.1282 (8) Å out of this plane.

In the crystal, the neutral binuclear complexes and nitrate ions are connected by classcial O—H···O and non-classical C—H···O hydrogen bonds into a three-dimensional framework (Fig. 2, Table 2).

### Experimental

A water solution (10ml) of Cu(NO_3_)_2_^.^3H_2_O (0.484g, 2mmol) was added slowly into a ethanol solution (10ml) containing N-benzyl-N'-(3-amino-3-dimethylpropyl)oxamide (1mmol, 0.262g) and sodium ethoxide (0.204 g, 3mmol). The mixture was stirred quickly for 2h, then an aqueous solution (5ml) of 1,10-phenanthroline (0.180 g, 1mmol) was added dropwise into the mixture. The reaction solution was heated at 303K with stirring for 12h. The resulting solution was filtered and the filtrate was kept at room temperature.  Green crystals suitable for X-ray analysis were obtained from the filtrate by slow evaporation for about three weeks. Yield, 69%, analysis, calculated for C_25_H_26_N_6_O_7_Cu_2_: C 46.22, H, 26.21; N 12.94%; found: C 46.26, H 26.29, N, 12.96%.

### Refinement

H atoms were positioned geometrically [0.93 (CH), 0.97 (CH_2_), 0.96 (CH_3_) and 0.84 (OH)Å] and constrained to ride on their parent atoms with *U*_iso_(H) =1.2(1.5 for methyl and hydroxy O)*U*_eq_(C/N).

### Crystal data

**Table:**

[Cu_2_(C_13_H_16_N_3_O_3_)(C_12_H_8_N_2_)(H_2_O)]NO_3_	*Z* = 2
*M**_r_* = 649.60	*F*(000) = 664
Triclinic, *P*1	*D*_x_ = 1.671 Mg m^−^^3^
Hall symbol: -P 1	Mo *K*α radiation, λ = 0.71073 Å
*a* = 10.543 (2) Å	Cell parameters from 3568 reflections
*b* = 11.070 (2) Å	θ = 2.5–26.1°
*c* = 11.404 (2) Å	µ = 1.71 mm^−^^1^
α = 89.88 (3)°	*T* = 296 K
β = 82.28 (3)°	Block, green
γ = 78.24 (3)°	0.56 × 0.51 × 0.46 mm
*V* = 1290.7 (4) Å^3^	

### Data collection

**Table:**

Bruker SMART CCD diffractometer	5984 independent reflections
Radiation source: fine-focus sealed tube	4281 reflections with *I* > 2σ(*I*)
graphite	*R*_int_ = 0.022
φ and ω scans	θ_max_ = 27.7°, θ_min_ = 2.0°
Absorption correction: multi-scan (*SADABS*; Sheldrick, 1996)	*h* = −13→13
*T*_min_ = 0.448, *T*_max_ = 0.507	*k* = −14→14
12596 measured reflections	*l* = −14→13

### Refinement

**Table:**

Refinement on *F*^2^	Primary atom site location: structure-invariant direct methods
Least-squares matrix: full	Secondary atom site location: difference Fourier map
*R*[*F*^2^ > 2σ(*F*^2^)] = 0.042	Hydrogen site location: inferred from neighbouring sites
*wR*(*F*^2^) = 0.129	H-atom parameters constrained
*S* = 1.00	*w* = 1/[σ^2^(*F*_o_^2^) + (0.0723*P*)^2^ + 0.4001*P*] where *P* = (*F*_o_^2^ + 2*F*_c_^2^)/3
5984 reflections	(Δ/σ)_max_ = 0.015
397 parameters	Δρ_max_ = 0.62 e Å^−^^3^
24 restraints	Δρ_min_ = −0.36 e Å^−^^3^

### Special details

**Table:**

Geometry. All esds (except the esd in the dihedral angle between two l.s. planes) are estimated using the full covariance matrix. The cell esds are taken into account individually in the estimation of esds in distances, angles and torsion angles; correlations between esds in cell parameters are only used when they are defined by crystal symmetry. An approximate (isotropic) treatment of cell esds is used for estimating esds involving l.s. planes.
Refinement. Refinement of F^2^ against ALL reflections. The weighted R-factor wR and goodness of fit S are based on F^2^, conventional R-factors R are based on F, with F set to zero for negative F^2^. The threshold expression of F^2^ > 2sigma(F^2^) is used only for calculating R-factors(gt) etc. and is not relevant to the choice of reflections for refinement. R-factors based on F^2^ are statistically about twice as large as those based on F, and R- factors based on ALL data will be even larger.

### Fractional atomic coordinates and isotropic or equivalent isotropic displacement parameters (Å^2^)

**Table:**

	*x*	*y*	*z*	*U*_iso_*/*U*_eq_	Occ. (<1)
Cu2	0.40128 (4)	0.40843 (4)	0.11469 (3)	0.04961 (14)	
Cu1	0.71425 (4)	0.40018 (3)	0.43073 (3)	0.04054 (13)	
O1	0.7295 (2)	0.5469 (2)	0.5175 (2)	0.0502 (6)	
O2	0.4700 (2)	0.5278 (2)	0.1980 (2)	0.0492 (5)	
O3	0.5323 (2)	0.2830 (2)	0.1794 (2)	0.0511 (6)	
O4	0.2339 (2)	0.3919 (2)	0.2581 (2)	0.0543 (6)	
H4A	0.2454	0.4047	0.3343	0.081*	
H4B	0.2222	0.3144	0.2533	0.081*	
N1	0.6041 (2)	0.5181 (2)	0.3446 (2)	0.0405 (6)	
N2	0.6665 (3)	0.2809 (2)	0.3246 (2)	0.0425 (6)	
N3	0.8428 (3)	0.2807 (2)	0.5112 (2)	0.0502 (7)	
N5	0.3419 (3)	0.2952 (3)	0.0091 (3)	0.0575 (8)	
N4	0.2882 (3)	0.5395 (3)	0.0326 (2)	0.0563 (8)	
C1	0.6697 (3)	0.6510 (3)	0.4709 (3)	0.0421 (7)	
C2	0.5972 (3)	0.6414 (3)	0.3765 (3)	0.0395 (6)	
C3	0.5358 (3)	0.7454 (3)	0.3233 (3)	0.0463 (7)	
H3	0.4893	0.7376	0.2609	0.056*	
C4	0.5437 (3)	0.8605 (3)	0.3631 (3)	0.0553 (9)	
H4	0.5030	0.9309	0.3276	0.066*	
C5	0.6123 (3)	0.8709 (3)	0.4561 (3)	0.0557 (9)	
H5	0.6157	0.9490	0.4839	0.067*	
C6	0.6760 (3)	0.7680 (3)	0.5090 (3)	0.0506 (8)	
H6	0.7233	0.7774	0.5704	0.061*	
C8	0.5473 (3)	0.4728 (3)	0.2668 (3)	0.0404 (7)	
C7	0.5848 (3)	0.3321 (3)	0.2560 (3)	0.0411 (7)	
C9	0.7093 (4)	0.1462 (3)	0.3177 (3)	0.0552 (9)	
H9A	0.7229	0.1188	0.2354	0.066*	0.50
H9B	0.6408	0.1092	0.3590	0.066*	0.50
H9C	0.7643	0.1220	0.2429	0.066*	0.50
H9D	0.6339	0.1081	0.3211	0.066*	0.50
C10A	0.8329 (7)	0.1021 (9)	0.3701 (7)	0.052 (2)	0.50
H10A	0.9037	0.1332	0.3250	0.063*	0.50
H10B	0.8554	0.0127	0.3657	0.063*	0.50
C11A	0.8168 (7)	0.1460 (5)	0.4988 (5)	0.0425 (14)	0.50
H11A	0.8772	0.0894	0.5405	0.051*	0.50
H11B	0.7287	0.1446	0.5357	0.051*	0.50
C12A	0.808 (2)	0.3076 (12)	0.6411 (7)	0.045 (3)	0.50
H12A	0.7188	0.3022	0.6651	0.067*	0.50
H12B	0.8195	0.3893	0.6585	0.067*	0.50
H12C	0.8642	0.2487	0.6832	0.067*	0.50
C13A	0.9744 (7)	0.2859 (10)	0.4700 (11)	0.070 (3)	0.50
H13A	0.9925	0.2667	0.3866	0.104*	0.50
H13B	1.0317	0.2271	0.5110	0.104*	0.50
H13C	0.9881	0.3673	0.4843	0.104*	0.50
C10B	0.7860 (8)	0.1011 (9)	0.4207 (9)	0.065 (3)	0.50
H10C	0.7232	0.1080	0.4920	0.078*	0.50
H10D	0.8228	0.0139	0.4062	0.078*	0.50
C11B	0.8951 (8)	0.1617 (7)	0.4474 (8)	0.070 (2)	0.50
H11C	0.9508	0.1075	0.4950	0.084*	0.50
H11D	0.9478	0.1750	0.3739	0.084*	0.50
C12B	0.807 (3)	0.2723 (13)	0.6375 (8)	0.051 (3)	0.50
H12D	0.7741	0.3537	0.6717	0.077*	0.50
H12E	0.8822	0.2333	0.6724	0.077*	0.50
H12F	0.7402	0.2244	0.6520	0.077*	0.50
C13B	0.9643 (8)	0.3396 (9)	0.4984 (10)	0.062 (3)	0.50
H13D	0.9932	0.3489	0.4160	0.093*	0.50
H13E	1.0330	0.2874	0.5333	0.093*	0.50
H13F	0.9424	0.4191	0.5379	0.093*	0.50
C14	0.3686 (4)	0.1724 (4)	0.0033 (4)	0.0696 (11)	
H14	0.4312	0.1293	0.0466	0.083*	
C15	0.3054 (5)	0.1069 (5)	−0.0656 (4)	0.0860 (14)	
H15	0.3252	0.0211	−0.0684	0.103*	
C16	0.2135 (5)	0.1706 (6)	−0.1293 (4)	0.0894 (16)	
H16	0.1708	0.1271	−0.1755	0.107*	
C17	0.1827 (4)	0.2987 (5)	−0.1264 (3)	0.0746 (13)	
C18	0.0877 (4)	0.3755 (7)	−0.1880 (4)	0.0936 (19)	
H18	0.0434	0.3380	−0.2379	0.112*	
C19	0.0609 (4)	0.4953 (7)	−0.1771 (4)	0.0887 (17)	
H19	−0.0022	0.5402	−0.2188	0.106*	
C20	0.1263 (4)	0.5612 (5)	−0.1017 (3)	0.0718 (13)	
C21	0.1012 (4)	0.6888 (5)	−0.0819 (4)	0.0835 (15)	
H21	0.0387	0.7396	−0.1201	0.100*	
C22	0.1681 (4)	0.7399 (5)	−0.0064 (4)	0.0789 (13)	
H22	0.1513	0.8248	0.0077	0.095*	
C23	0.2617 (4)	0.6610 (4)	0.0485 (4)	0.0669 (11)	
H23	0.3079	0.6953	0.0989	0.080*	
C24	0.2208 (3)	0.4897 (4)	−0.0416 (3)	0.0608 (10)	
C25	0.2498 (3)	0.3585 (4)	−0.0538 (3)	0.0597 (10)	
N6	0.1811 (5)	0.0538 (5)	0.2109 (4)	0.0969 (13)	
O5	0.1519 (4)	0.1668 (4)	0.2262 (4)	0.1159 (13)	
O6	0.2803 (6)	−0.0114 (7)	0.2088 (7)	0.234 (4)	
O7	0.0940 (7)	0.0076 (5)	0.1804 (5)	0.172 (2)	

### Atomic displacement parameters (Å^2^)

**Table:**

	*U*^11^	*U*^22^	*U*^33^	*U*^12^	*U*^13^	*U*^23^
Cu2	0.0456 (2)	0.0637 (3)	0.0430 (2)	−0.01150 (19)	−0.01790 (17)	−0.00329 (19)
Cu1	0.0434 (2)	0.0357 (2)	0.0437 (2)	−0.00387 (15)	−0.01655 (16)	−0.00239 (15)
O1	0.0609 (14)	0.0401 (12)	0.0532 (13)	−0.0054 (10)	−0.0281 (11)	−0.0036 (10)
O2	0.0479 (12)	0.0504 (13)	0.0528 (13)	−0.0077 (10)	−0.0228 (10)	0.0007 (10)
O3	0.0535 (13)	0.0517 (14)	0.0529 (14)	−0.0131 (11)	−0.0210 (11)	−0.0075 (11)
O4	0.0598 (14)	0.0626 (15)	0.0434 (12)	−0.0153 (12)	−0.0127 (10)	−0.0018 (11)
N1	0.0398 (13)	0.0376 (13)	0.0459 (15)	−0.0055 (10)	−0.0163 (11)	0.0016 (11)
N2	0.0496 (15)	0.0349 (13)	0.0462 (15)	−0.0094 (11)	−0.0168 (12)	−0.0026 (11)
N3	0.0547 (16)	0.0449 (15)	0.0490 (16)	0.0026 (12)	−0.0199 (13)	−0.0062 (12)
N5	0.0494 (16)	0.083 (2)	0.0421 (16)	−0.0157 (15)	−0.0117 (13)	−0.0098 (15)
N4	0.0475 (16)	0.084 (2)	0.0384 (15)	−0.0123 (15)	−0.0112 (12)	0.0075 (15)
C1	0.0408 (16)	0.0395 (16)	0.0465 (17)	−0.0064 (13)	−0.0102 (13)	−0.0017 (13)
C2	0.0394 (15)	0.0363 (15)	0.0434 (17)	−0.0076 (12)	−0.0081 (13)	−0.0011 (12)
C3	0.0425 (16)	0.0457 (18)	0.0501 (19)	−0.0043 (14)	−0.0120 (14)	0.0023 (14)
C4	0.056 (2)	0.0373 (17)	0.069 (2)	−0.0013 (15)	−0.0087 (17)	0.0053 (16)
C5	0.057 (2)	0.0368 (17)	0.072 (2)	−0.0082 (15)	−0.0089 (18)	−0.0062 (16)
C6	0.0534 (19)	0.0430 (18)	0.058 (2)	−0.0090 (15)	−0.0174 (16)	−0.0075 (15)
C8	0.0361 (15)	0.0442 (17)	0.0433 (17)	−0.0108 (12)	−0.0101 (12)	0.0020 (13)
C7	0.0400 (15)	0.0436 (17)	0.0420 (17)	−0.0124 (13)	−0.0079 (13)	−0.0021 (13)
C9	0.070 (2)	0.0364 (17)	0.062 (2)	−0.0106 (16)	−0.0221 (18)	−0.0026 (15)
C10A	0.039 (4)	0.037 (4)	0.079 (6)	−0.004 (4)	−0.007 (4)	−0.005 (4)
C11A	0.037 (3)	0.039 (3)	0.052 (4)	−0.002 (3)	−0.016 (3)	0.006 (3)
C12A	0.052 (5)	0.035 (8)	0.048 (5)	−0.006 (6)	−0.011 (4)	0.007 (3)
C13A	0.039 (4)	0.079 (7)	0.078 (8)	0.011 (4)	0.002 (4)	−0.019 (5)
C10B	0.041 (5)	0.039 (4)	0.118 (9)	−0.007 (4)	−0.023 (5)	0.011 (6)
C11B	0.061 (5)	0.066 (5)	0.078 (6)	0.003 (4)	−0.015 (4)	−0.020 (4)
C12B	0.063 (6)	0.032 (8)	0.063 (6)	−0.014 (7)	−0.016 (4)	0.008 (4)
C13B	0.039 (4)	0.082 (7)	0.050 (6)	0.016 (4)	0.008 (3)	0.001 (5)
C14	0.068 (2)	0.085 (3)	0.059 (2)	−0.019 (2)	−0.0169 (19)	−0.014 (2)
C15	0.087 (3)	0.103 (4)	0.073 (3)	−0.029 (3)	−0.015 (3)	−0.029 (3)
C16	0.084 (3)	0.136 (5)	0.059 (3)	−0.046 (3)	−0.012 (2)	−0.031 (3)
C17	0.054 (2)	0.133 (4)	0.040 (2)	−0.027 (2)	−0.0074 (17)	−0.017 (2)
C18	0.053 (2)	0.192 (6)	0.041 (2)	−0.032 (3)	−0.0163 (19)	−0.016 (3)
C19	0.051 (2)	0.170 (6)	0.044 (2)	−0.014 (3)	−0.0178 (18)	0.008 (3)
C20	0.047 (2)	0.126 (4)	0.038 (2)	−0.009 (2)	−0.0063 (15)	0.016 (2)
C21	0.058 (2)	0.125 (4)	0.057 (3)	0.004 (3)	−0.007 (2)	0.036 (3)
C22	0.068 (3)	0.098 (4)	0.064 (3)	−0.005 (2)	−0.005 (2)	0.026 (2)
C23	0.064 (2)	0.081 (3)	0.054 (2)	−0.011 (2)	−0.0093 (18)	0.014 (2)
C24	0.0444 (18)	0.106 (3)	0.0308 (17)	−0.0124 (19)	−0.0050 (14)	0.0052 (18)
C25	0.0413 (18)	0.106 (3)	0.0328 (17)	−0.0168 (19)	−0.0052 (14)	−0.0071 (18)
N6	0.102 (4)	0.099 (3)	0.088 (3)	−0.015 (3)	−0.013 (3)	0.020 (3)
O5	0.126 (3)	0.112 (3)	0.111 (3)	−0.044 (3)	0.009 (2)	−0.024 (3)
O6	0.127 (4)	0.271 (7)	0.272 (7)	0.036 (4)	−0.028 (5)	0.155 (6)
O7	0.185 (5)	0.153 (5)	0.188 (5)	−0.061 (4)	−0.023 (4)	−0.042 (4)

### Geometric parameters (Å, °)

**Table:**

Cu2—O2	1.938 (2)	C10A—H10A	0.9700
Cu2—O3	1.967 (2)	C10A—H10B	0.9700
Cu2—N5	1.986 (3)	C11A—H11A	0.9700
Cu2—N4	1.998 (3)	C11A—H11B	0.9700
Cu2—O4	2.275 (2)	C12A—H12A	0.9600
Cu1—N1	1.924 (3)	C12A—H12B	0.9600
Cu1—O1	1.950 (2)	C12A—H12C	0.9600
Cu1—N2	1.976 (2)	C13A—H13A	0.9600
Cu1—N3	2.007 (3)	C13A—H13B	0.9600
O1—C1	1.341 (4)	C13A—H13C	0.9600
O2—C8	1.272 (4)	C10B—C11B	1.508 (8)
O3—C7	1.273 (4)	C10B—H10C	0.9700
O4—H4A	0.9085	C10B—H10D	0.9700
O4—H4B	0.8938	C11B—H11C	0.9700
N1—C8	1.289 (4)	C11B—H11D	0.9700
N1—C2	1.398 (4)	C12B—H12D	0.9600
N2—C7	1.286 (4)	C12B—H12E	0.9600
N2—C9	1.467 (4)	C12B—H12F	0.9600
N3—C13A	1.416 (7)	C13B—H13D	0.9600
N3—C12B	1.446 (8)	C13B—H13E	0.9600
N3—C11B	1.472 (6)	C13B—H13F	0.9600
N3—C12A	1.493 (8)	C14—C15	1.388 (5)
N3—C13B	1.542 (8)	C14—H14	0.9300
N3—C11A	1.580 (6)	C15—C16	1.368 (7)
N5—C14	1.331 (5)	C15—H15	0.9300
N5—C25	1.362 (5)	C16—C17	1.388 (7)
N4—C23	1.325 (5)	C16—H16	0.9300
N4—C24	1.359 (5)	C17—C25	1.406 (5)
C1—C6	1.385 (4)	C17—C18	1.435 (7)
C1—C2	1.418 (4)	C18—C19	1.301 (8)
C2—C3	1.383 (4)	C18—H18	0.9300
C3—C4	1.376 (4)	C19—C20	1.454 (7)
C3—H3	0.9300	C19—H19	0.9300
C4—C5	1.379 (5)	C20—C21	1.396 (7)
C4—H4	0.9300	C20—C24	1.396 (5)
C5—C6	1.381 (5)	C21—C22	1.374 (7)
C5—H5	0.9300	C21—H21	0.9300
C6—H6	0.9300	C22—C23	1.394 (6)
C8—C7	1.528 (4)	C22—H22	0.9300
C9—C10A	1.497 (7)	C23—H23	0.9300
C9—C10B	1.539 (8)	C24—C25	1.425 (6)
C9—H9A	0.9700	N6—O6	1.140 (6)
C9—H9B	0.9700	N6—O7	1.227 (6)
C9—H9C	0.9700	N6—O5	1.232 (6)
C9—H9D	0.9700	N6—O5	1.232 (6)
C10A—C11A	1.523 (8)		
			
O2—Cu2—O3	85.77 (9)	H9A—C9—H9D	82.9
O2—Cu2—N5	172.13 (11)	H9C—C9—H9D	108.1
O3—Cu2—N5	96.96 (12)	C9—C10A—C11A	110.7 (5)
O2—Cu2—N4	92.76 (12)	C9—C10A—H10A	109.5
O3—Cu2—N4	172.21 (10)	C11A—C10A—H10A	109.5
N5—Cu2—N4	83.54 (14)	C9—C10A—H10B	109.5
O2—Cu2—O4	96.91 (9)	C11A—C10A—H10B	109.5
O3—Cu2—O4	95.19 (10)	H10A—C10A—H10B	108.1
N5—Cu2—O4	90.21 (11)	C10A—C11A—N3	112.4 (5)
N4—Cu2—O4	92.58 (10)	C10A—C11A—H11A	109.1
N1—Cu1—O1	83.22 (10)	N3—C11A—H11A	109.1
N1—Cu1—N2	82.68 (11)	C10A—C11A—H11B	109.1
O1—Cu1—N2	165.80 (10)	N3—C11A—H11B	109.1
N1—Cu1—N3	174.91 (11)	H11A—C11A—H11B	107.9
O1—Cu1—N3	96.11 (10)	N3—C12A—H12A	109.5
N2—Cu1—N3	98.09 (11)	N3—C12A—H12B	109.5
C1—O1—Cu1	111.94 (19)	H12A—C12A—H12B	109.5
C8—O2—Cu2	110.0 (2)	N3—C12A—H12C	109.5
C7—O3—Cu2	110.2 (2)	H12A—C12A—H12C	109.5
Cu2—O4—H4A	117.3	H12B—C12A—H12C	109.5
Cu2—O4—H4B	106.6	N3—C13A—H13A	109.5
H4A—O4—H4B	106.1	N3—C13A—H13B	109.5
C8—N1—C2	129.5 (3)	H13A—C13A—H13B	109.5
C8—N1—Cu1	116.0 (2)	N3—C13A—H13C	109.5
C2—N1—Cu1	114.5 (2)	H13A—C13A—H13C	109.5
C7—N2—C9	118.3 (3)	H13B—C13A—H13C	109.5
C7—N2—Cu1	113.2 (2)	C11B—C10B—C9	120.0 (7)
C9—N2—Cu1	128.5 (2)	C11B—C10B—H10C	107.3
C13A—N3—C12B	117.5 (12)	C9—C10B—H10C	107.3
C13A—N3—C11B	76.3 (5)	C11B—C10B—H10D	107.3
C12B—N3—C11B	115.1 (7)	C9—C10B—H10D	107.3
C13A—N3—C12A	112.4 (10)	H10C—C10B—H10D	106.9
C11B—N3—C12A	129.6 (7)	N3—C11B—C10B	111.0 (7)
C12B—N3—C13B	104.9 (10)	N3—C11B—H11C	109.4
C11B—N3—C13B	101.5 (4)	C10B—C11B—H11C	109.4
C12A—N3—C13B	94.9 (9)	N3—C11B—H11D	109.4
C13A—N3—C11A	111.8 (5)	C10B—C11B—H11D	109.4
C12B—N3—C11A	87.3 (6)	H11C—C11B—H11D	108.0
C12A—N3—C11A	102.6 (5)	N3—C12B—H12D	109.5
C13B—N3—C11A	135.8 (4)	N3—C12B—H12E	109.5
C13A—N3—Cu1	112.8 (6)	H12D—C12B—H12E	109.5
C12B—N3—Cu1	114.8 (10)	N3—C12B—H12F	109.5
C11B—N3—Cu1	114.9 (4)	H12D—C12B—H12F	109.5
C12A—N3—Cu1	106.9 (9)	H12E—C12B—H12F	109.5
C13B—N3—Cu1	103.2 (5)	N3—C13B—H13D	109.5
C11A—N3—Cu1	109.7 (3)	N3—C13B—H13E	109.5
C14—N5—C25	119.3 (3)	H13D—C13B—H13E	109.5
C14—N5—Cu2	129.2 (3)	N3—C13B—H13F	109.5
C25—N5—Cu2	111.0 (3)	H13D—C13B—H13F	109.5
C23—N4—C24	118.1 (4)	H13E—C13B—H13F	109.5
C23—N4—Cu2	130.2 (3)	N5—C14—C15	121.7 (4)
C24—N4—Cu2	111.3 (3)	N5—C14—H14	119.2
O1—C1—C6	123.6 (3)	C15—C14—H14	119.2
O1—C1—C2	118.6 (3)	C16—C15—C14	119.0 (5)
C6—C1—C2	117.8 (3)	C16—C15—H15	120.5
C3—C2—N1	127.4 (3)	C14—C15—H15	120.5
C3—C2—C1	121.2 (3)	C15—C16—C17	121.4 (4)
N1—C2—C1	111.4 (3)	C15—C16—H16	119.3
C4—C3—C2	119.6 (3)	C17—C16—H16	119.3
C4—C3—H3	120.2	C16—C17—C25	116.4 (4)
C2—C3—H3	120.2	C16—C17—C18	126.5 (5)
C3—C4—C5	119.7 (3)	C25—C17—C18	117.1 (5)
C3—C4—H4	120.2	C19—C18—C17	122.8 (5)
C5—C4—H4	120.2	C19—C18—H18	118.6
C4—C5—C6	121.4 (3)	C17—C18—H18	118.6
C4—C5—H5	119.3	C18—C19—C20	122.1 (5)
C6—C5—H5	119.3	C18—C19—H19	118.9
C5—C6—C1	120.2 (3)	C20—C19—H19	118.9
C5—C6—H6	119.9	C21—C20—C24	117.1 (4)
C1—C6—H6	119.9	C21—C20—C19	126.1 (5)
O2—C8—N1	129.6 (3)	C24—C20—C19	116.8 (5)
O2—C8—C7	117.3 (3)	C22—C21—C20	120.5 (4)
N1—C8—C7	113.0 (3)	C22—C21—H21	119.7
O3—C7—N2	129.5 (3)	C20—C21—H21	119.7
O3—C7—C8	115.4 (3)	C21—C22—C23	118.1 (5)
N2—C7—C8	115.1 (3)	C21—C22—H22	121.0
N2—C9—C10A	112.8 (4)	C23—C22—H22	121.0
N2—C9—C10B	110.3 (5)	N4—C23—C22	123.4 (4)
N2—C9—H9A	109.0	N4—C23—H23	118.3
C10A—C9—H9A	109.0	C22—C23—H23	118.3
C10B—C9—H9A	131.0	N4—C24—C20	122.8 (4)
N2—C9—H9B	109.0	N4—C24—C25	116.4 (3)
C10A—C9—H9B	109.0	C20—C24—C25	120.9 (4)
C10B—C9—H9B	85.6	N5—C25—C17	122.3 (4)
H9A—C9—H9B	107.8	N5—C25—C24	117.4 (3)
N2—C9—H9C	109.4	C17—C25—C24	120.3 (4)
C10A—C9—H9C	84.3	O6—N6—O7	115.5 (7)
C10B—C9—H9C	109.7	O6—N6—O5	129.4 (7)
H9B—C9—H9C	129.6	O7—N6—O5	114.5 (6)
N2—C9—H9D	109.7	O6—N6—O5	129.4 (7)
C10A—C9—H9D	128.2	O7—N6—O5	114.5 (6)
C10B—C9—H9D	109.5		
			
N1—Cu1—O1—C1	−5.2 (2)	Cu1—N2—C7—O3	−178.5 (3)
N2—Cu1—O1—C1	−12.1 (5)	C9—N2—C7—C8	−178.8 (3)
N3—Cu1—O1—C1	169.7 (2)	Cu1—N2—C7—C8	2.6 (3)
O3—Cu2—O2—C8	−10.2 (2)	O2—C8—C7—O3	−1.2 (4)
N4—Cu2—O2—C8	177.5 (2)	N1—C8—C7—O3	−179.3 (2)
O4—Cu2—O2—C8	84.6 (2)	O2—C8—C7—N2	177.8 (2)
O2—Cu2—O3—C7	9.6 (2)	N1—C8—C7—N2	−0.3 (4)
N5—Cu2—O3—C7	−177.8 (2)	C7—N2—C9—C10A	161.3 (4)
O4—Cu2—O3—C7	−87.0 (2)	Cu1—N2—C9—C10A	−20.4 (5)
O1—Cu1—N1—C8	−175.3 (2)	C7—N2—C9—C10B	−169.8 (4)
N2—Cu1—N1—C8	3.0 (2)	Cu1—N2—C9—C10B	8.5 (6)
O1—Cu1—N1—C2	4.5 (2)	N2—C9—C10A—C11A	57.4 (8)
N2—Cu1—N1—C2	−177.2 (2)	C10B—C9—C10A—C11A	−33.0 (10)
N1—Cu1—N2—C7	−3.1 (2)	C9—C10A—C11A—N3	−84.7 (7)
O1—Cu1—N2—C7	3.8 (6)	C13A—N3—C11A—C10A	−65.0 (8)
N3—Cu1—N2—C7	−178.0 (2)	C12B—N3—C11A—C10A	176.4 (12)
N1—Cu1—N2—C9	178.6 (3)	C11B—N3—C11A—C10A	−44.5 (7)
O1—Cu1—N2—C9	−174.5 (4)	C12A—N3—C11A—C10A	174.4 (11)
N3—Cu1—N2—C9	3.6 (3)	C13B—N3—C11A—C10A	−74.9 (9)
O1—Cu1—N3—C13A	−76.3 (5)	Cu1—N3—C11A—C10A	61.0 (5)
N2—Cu1—N3—C13A	104.2 (5)	N2—C9—C10B—C11B	−49.2 (10)
O1—Cu1—N3—C12B	62.0 (8)	C10A—C9—C10B—C11B	51.2 (10)
N2—Cu1—N3—C12B	−117.6 (7)	C13A—N3—C11B—C10B	−163.7 (9)
O1—Cu1—N3—C11B	−161.2 (4)	C12B—N3—C11B—C10B	82.0 (13)
N2—Cu1—N3—C11B	19.3 (4)	C12A—N3—C11B—C10B	88.3 (14)
O1—Cu1—N3—C12A	47.8 (7)	C13B—N3—C11B—C10B	−165.3 (8)
N2—Cu1—N3—C12A	−131.8 (7)	C11A—N3—C11B—C10B	35.8 (6)
O1—Cu1—N3—C13B	−51.6 (4)	Cu1—N3—C11B—C10B	−54.7 (8)
N2—Cu1—N3—C13B	128.9 (4)	C9—C10B—C11B—N3	77.8 (11)
O1—Cu1—N3—C11A	158.3 (3)	C25—N5—C14—C15	0.2 (6)
N2—Cu1—N3—C11A	−21.2 (3)	Cu2—N5—C14—C15	171.6 (3)
O3—Cu2—N5—C14	10.2 (3)	N5—C14—C15—C16	0.1 (7)
N4—Cu2—N5—C14	−177.6 (3)	C14—C15—C16—C17	0.1 (7)
O4—Cu2—N5—C14	−85.0 (3)	C15—C16—C17—C25	−0.7 (6)
O3—Cu2—N5—C25	−177.9 (2)	C15—C16—C17—C18	−179.3 (4)
N4—Cu2—N5—C25	−5.7 (2)	C16—C17—C18—C19	177.3 (5)
O4—Cu2—N5—C25	86.9 (2)	C25—C17—C18—C19	−1.4 (7)
O2—Cu2—N4—C23	−8.5 (3)	C17—C18—C19—C20	0.5 (8)
N5—Cu2—N4—C23	178.5 (3)	C18—C19—C20—C21	−177.9 (4)
O4—Cu2—N4—C23	88.6 (3)	C18—C19—C20—C24	0.4 (6)
O2—Cu2—N4—C24	178.7 (2)	C24—C20—C21—C22	0.3 (6)
N5—Cu2—N4—C24	5.7 (2)	C19—C20—C21—C22	178.6 (4)
O4—Cu2—N4—C24	−84.2 (2)	C20—C21—C22—C23	0.5 (6)
Cu1—O1—C1—C6	−173.9 (3)	C24—N4—C23—C22	0.3 (5)
Cu1—O1—C1—C2	5.2 (3)	Cu2—N4—C23—C22	−172.1 (3)
C8—N1—C2—C3	−6.4 (5)	C21—C22—C23—N4	−0.8 (6)
Cu1—N1—C2—C3	173.9 (3)	C23—N4—C24—C20	0.5 (5)
C8—N1—C2—C1	176.8 (3)	Cu2—N4—C24—C20	174.3 (3)
Cu1—N1—C2—C1	−2.9 (3)	C23—N4—C24—C25	−178.4 (3)
O1—C1—C2—C3	−178.6 (3)	Cu2—N4—C24—C25	−4.7 (4)
C6—C1—C2—C3	0.6 (4)	C21—C20—C24—N4	−0.8 (5)
O1—C1—C2—N1	−1.7 (4)	C19—C20—C24—N4	−179.3 (3)
C6—C1—C2—N1	177.5 (3)	C21—C20—C24—C25	178.1 (3)
N1—C2—C3—C4	−177.1 (3)	C19—C20—C24—C25	−0.4 (5)
C1—C2—C3—C4	−0.7 (5)	C14—N5—C25—C17	−0.9 (5)
C2—C3—C4—C5	−0.3 (5)	Cu2—N5—C25—C17	−173.7 (3)
C3—C4—C5—C6	1.4 (5)	C14—N5—C25—C24	177.7 (3)
C4—C5—C6—C1	−1.5 (5)	Cu2—N5—C25—C24	4.9 (4)
O1—C1—C6—C5	179.6 (3)	C16—C17—C25—N5	1.1 (5)
C2—C1—C6—C5	0.5 (5)	C18—C17—C25—N5	179.9 (3)
Cu2—O2—C8—N1	−173.3 (3)	C16—C17—C25—C24	−177.5 (4)
Cu2—O2—C8—C7	9.0 (3)	C18—C17—C25—C24	1.3 (5)
C2—N1—C8—O2	0.2 (5)	N4—C24—C25—N5	−0.1 (5)
Cu1—N1—C8—O2	179.9 (3)	C20—C24—C25—N5	−179.1 (3)
C2—N1—C8—C7	177.9 (3)	N4—C24—C25—C17	178.5 (3)
Cu1—N1—C8—C7	−2.4 (3)	C20—C24—C25—C17	−0.5 (5)
Cu2—O3—C7—N2	174.0 (3)	O6—N6—O5—O5	0.0 (3)
Cu2—O3—C7—C8	−7.2 (3)	O7—N6—O5—O5	0.00 (17)
C9—N2—C7—O3	0.0 (5)		

### Hydrogen-bond geometry (Å, °)

**Table:**

*D*—H···*A*	*D*—H	H···*A*	*D*···*A*	*D*—H···*A*
O4—H4A···O1^i^	0.91	1.84	2.745 (3)	172
O4—H4B···O5	0.89	1.97	2.839 (5)	164
C19—H19···O4^ii^	0.93	2.51	3.355 (5)	152
C21—H21···O5^ii^	0.93	2.53	3.437 (7)	167

Symmetry codes: (i) −*x*+1, −*y*+1, −*z*+1;  (ii) −*x*, −*y*+1, −*z*.

## References

[bb1] Bruker (1998). *SMART* and *SAINT* Bruker AXS Inc., Madison, Wisconsin, USA.

[bb2] Kou, H. Z., Zhou, B. C., Gao, S. & Wang, R. J. (1999). *Angew. Chem. Int. Ed.* **42**, 3288–3291. 10.1002/anie.200351248 12876746

[bb3] Ojima, H. & Nonoyama, K. (1988). *Coord. Chem. Rev.* **92**, 85–92.

[bb4] Sheldrick, G. M. (1996). *SADABS* University of Göttingen, Germany.

[bb5] Sheldrick, G. M. (2008). *Acta Cryst.* A**64**, 112–122. 10.1107/S0108767307043930 18156677

[bb6] Spek, A. L. (2009). *Acta Cryst.* D**65**, 148–155. 10.1107/S090744490804362X PMC2631630 19171970

[bb7] Wang, S. B., Yang, G. M., Yu, L. H., Wang, Q. L. & Liao, D. Z. (2003). *Transition Met. Chem* **28**, 632–634.

